# Verbal Abuse, Depersonalization, and the Innate Alarm and Defensive Systems: A Single Case Illustration of Treatment with Deep Brain Reorienting

**DOI:** 10.1007/s40653-024-00672-z

**Published:** 2024-12-06

**Authors:** Costanzo Frau, Frank M. Corrigan

**Affiliations:** 1 Manchester Metropolitan University (MMU), Psychotherapy Service and Research On Trauma & Dissociation, Via Dante 52, 09121 Cagliari, Italy; 2 Trauma Psychotherapy Scotland, 13 Fitzroy Place, Finnieston, Glasgow, G3 7RW UK

**Keywords:** Trauma, Verbal abuse, Dissociation, Depersonalization, Innate alarm system, Deep brain reorienting

## Abstract

This study aimed to a) discuss the neurobiological mechanisms of depersonalization as arising from activation at the brainstem level and b) assess the effectiveness of deep brain reorienting psychotherapy (DBR) with a patient presenting with depersonalization-derealization disorder (DDD). In the first part of the paper, we discuss verbal abuse as a severe form of relational trauma and how it can be connected to depersonalization. It is argued that suddenly aversive experiences engage the brainstem locus coeruleus in widespread noradrenergic activation of the thalamus and cortex such that the balance of functioning within the cortex becomes disturbed and a subjective experience of chronic depersonalization results. In the second part, the single-case study aims to provide initial evidence of how the patient experienced and responded to DBR therapy. Pre- and post-treatment measures consisted of instruments to measure depersonalization, social anxiety symptoms and quality of life. After 43 DBR sessions, the participant's depersonalization and comorbid symptoms decreased significantly. Patients with DDD may benefit from DBR. Future research is required to address generalizability to a larger population.

## Introduction

Although sexual and physical abuse are the more overt forms of childhood adversity that have been studied, there has recently been greater attention given to other forms of emotional maltreatment, such as witnessing domestic violence and receiving verbal abuse (Cancilliere et al., 2022; Dutra et al., 2009; Teicher et al., 2006). Maternal verbal abuse seems to be correlated with borderline, narcissistic, obsessive–compulsive, and paranoid personality disorders, even after controlling for other variables (Johnson et al., 2001, as cited in Teicher et al., 2006).

Moreover, research has revealed that emotional abuse is a predictor of depersonalization disorder and "may play an important role in the genesis of depersonalization symptoms" (Simeon et al., 2001; p. 1032).

Depersonalization symptoms are common to different diagnoses, such as panic disorder (Hunter et al., 2004), depression (Soffer-Dudek, 2014), bipolar affective disorder (Mula et al., 2009) and PTSD (van Huijstee & Vermetten, 2018).

Instead, depersonalization disorder, defined as "depersonalization-derealization disorder" (DDD) in the DSM-5 (American Psychiatric Association, 2013), is a syndrome characterized by four clusters of symptoms: anomalous body experiences, emotional numbing, anomalous subjective recall, and alienation from surroundings (Sierra et al., 2005, as cited in Sierra, 2009). Depersonalization disorder is often seen as being on a spectrum of dissociative disorders that are considered to arise from early emotional trauma, including adverse attachment experiences (Dutra et al., 2009; Farina et al., 2019; Lyons-Ruth et al., 2006). Despite the disorder having been considered rare, with health service professionals tending to make incorrect diagnoses, for many years (Hunter et al., 2003), some studies have defined a prevalence of pathological depersonalization between 1% and 2.4% in the general population (Kate et al., 2020; Michal et al., 2009; Simeon & Abugel, 2006; Yang et al., 2022). In addition, specific structural and functional brain alterations of this potentially intractable disorder have also been demonstrated (Daniels et al., 2015; Salami et al., 2020).

In this paper, depersonalization is considered to be a consequence of parental verbal abuse. How this theoretical framework supports the deep brain reorienting (DBR) technique used with a complex DDD is described.

Here, it is argued that there is a specific kind of emotional shock trauma that causes immediate disturbance of the subjective sense of the self in the body and in relation to the world around. This shock, especially if repeated, could establish a chronic sense of estrangement from the body and the surroundings, both of which could be experienced as numbness of sensory perceptions. Chronic and severe verbal abuse may be experienced as emotionally shocking and lead to a state of continuous depersonalization, a cortical adaptation to traumatic experiences, or an epiphenomenon of the brainstem’s responses to them, which continues long after the initial stimuli have been withdrawn from the environment. The last part of the paper will focus on the treatment of a single case of depersonalization disorder that had arisen from an early life in which verbal abuse was prominent. The trauma therapy used is DBR, a modality that pays particular attention to pre-affective shock experiences (Corrigan & Christie-Sands, 2020).

## Verbal Abuse as a Severe Form of Relational Trauma

The concept of childhood trauma, as generally defined in the psychological literature, refers to physical abuse, sexual abuse, and emotional abuse. Emotional abuse can include emotional neglect, emotional manipulation, witnessing domestic violence and verbal abuse (Teicher & Samson, 2013). Parental verbal aggression is considered a serious form of maltreatment that strongly correlates with dissociative symptoms. Indeed, Dutra and colleagues (2009) found that the only specific form of childhood trauma strongly related to dissociative symptoms was severe verbal abuse.

A recent study of the association between verbal abuse during pregnancy and the rate of referral for screening of hearing in newborns suggested a correlation. The authors proposed that development of auditory function in the fetal brain is impaired when the loud voice of the mother’s intimate partner coincides with the mother’s stress response (Komori et al., 2019).

Teicher and Samson (2013) described verbal aggression as "communications intended to inflict intense humiliation, denigration or extreme fear" (Teicher & Samson, 2013, p. 21), and this severe form of interpersonal trauma may have direct harmful effects on brain development (Teicher & Samson, 2016; Choi et al., 2009; Tomoda et al., 2011; Kim et al., 2019; Teicher et al., 2006, 2016).

Parental verbal aggression can simultaneously activate the defense system and the attachment system of the child, creating a conflict between approach toward and flight from the caregiver (Liotti, 2017). Nevertheless, in many cases, verbal abuse may not involve a long-lasting activation of the defense system but take the form of a finer attack on the formation of the identity of the child who can experience a strong sense of inadequacy and guilt in this phase of development (Erikson, 1959; Steinberg & Schnall, 2001).

Verbal abuse may be an instrument for the definition of social ranking in the hierarchies of dominance-submission (as described in, for example, Gilbert, 2017; Lichtenberg et al., 2011; Liotti, 2017), and verbally abusive parents may continuously keep their child in the submission ranking subroutine. Consequently, the emotion of shame, connected to it and perceived at the level of primary consciousness, is over-experienced and could interfere with the child’s development and influence later interactions with peers (Mills et al., 2015; Mills, 2005; Bennet et al., 2010).

As has been argued by Dutra et al. (2009), verbal abuse in children with poor early attachment experiences can affect the internalized representation of self negatively and impact the self-other relationship. Indeed, in defining a sense of self, the child tends to identify himself with the parental figures and to introject them (Erikson, 1959; Schore, 1998). Shame that emerges from this introjected state is related to social anxiety and depression (Gilbert, 2000) and can be a central emotion in complex posttraumatic stress and dissociative disorders (Dorahy et al., 2015).

## The Neurobiological Consequences of Verbal Abuse: Hyperactivation of the Innate Alarm System and the Depersonalization Process

The hypothesis proposing shock as an aetiological factor in DPD could be seen as representing depersonalization, at least acutely, as an epiphenomenon rather than an adaptation. Here, it is proposed that the depersonalization symptom is the result of activation of ascending systems from the brainstem in response to an adverse event. When something is shocking or horrifying, the brainstem arouses the upper brain through ascending systems such as that from the locus coeruleus (Corrigan & Christie-Sands, 2020; Corrigan et al., 2023). The noradrenergic arousal alters the balance within the cortex, perhaps through an impact on the retrosplenial cortex, an area demonstrated in animal models to be critical in navigating to safety (Campagner et al., 2023) and in dissociative responses (Jovasevic et al., 2015; Vesuna et al., 2020). The basis of attachment is hypothesized to be in the connection system that mediates the sensorimotor transformation of relational, interactional stimuli. That is, the superior colliculi (SC) in the midbrain of the child will respond first to any verbal abuse, and an approach/defence conflict can occur at this level. Before there is any affective or defensive response mediated by the midbrain periaqueductal gray (PAG), a shocking stimulus can activate an innate alarm system (Corrigan & Christie-Sands, 2020). This comes online when the registration of a horrifying stimulus in the superior colliculus (SC) rapidly elicits involvement of the locus coeruleus through a circuit also including the thalamus and the amygdala (Liddell et al., 2005).

Therefore, sudden, and unexpectedly vituperative verbal abuse may engage the innate alarm system. The registration in the SC of the angry face and the critical tone and volume of the words activate the locus coeruleus in the upper pons. In turn, this produces widespread activation of the body and the brain, with changes in arousal, attention, and gating of sensory stimuli (Samuels & Szabadi, 2008). It is hypothesised that ascending projections, via the thalamus, to the cortex initiate a disturbance in the balance of cortical functioning such that there is a loss of a sense of continuity within the self, the body, and the surrounding space, producing a subjective sense of depersonalization.

Acoustic processing is the specialization of the inferior, rather than the superior, colliculi (e.g., Oberle et al., 2022), and projections from the auditory cortex to the inferior colliculi (IC) mediate innate, sound-induced flight behavior through subsequent projections from the IC to the dorsal PAG (Xiong et al., 2015). The auditory cortex can amplify responses through its influence on the IC or provoke responses through onwards transmission from the IC to the PAG. Increased vigilance to threat may be mediated not only by the locus coeruleus projections to the superior colliculi (Li et al., 2018) but also by a sensitization of the inferior colliculi when verbal abuse and threats have been traumatizing. There are close functional links between the SC and the IC for the localization of auditory stimuli, and the IC projects to the intermediate layers of the SC, the structure we have focused on for the immediate impact of shock and horror.

In this hypothesis, verbal abuse is a kind of relational trauma that elicits brainstem activation through the superior and inferior colliculi (SC & IC). These would then activate shock and horror responses (Corrigan & Christie-Sands, 2020) through the innate alarm circuit (see Liddell et al., 2005), which includes the locus coeruleus and the amygdala, before the engagement of the affective and defensive responses mediated by the PAG (Bandler et al., 2000; Panksepp, 1998). The intracortical disequilibrium resulting from the stimulation of ascending projections from the brainstem is subjectively experienced as depersonalization and can become a chronic disorder. It may not only be the noradrenergic projections from the locus coeruleus that contribute to shock, horror, and other unpleasant arousal responses. Cholinergic projections from the midbrain reticular formation activating limbic and prefrontal cortical circuits may also be involved (e.g., Terpou et al., 2019). Thus, there is scope for variable activation of thalamic and cortical areas in response to subliminal threat-related stimuli, which may be subjectively experienced as depersonalization and/or derealization.

In summary, we propose that adversity sufficiently intense that it leads to limbic learning and disruption of normally integrated cortical functioning necessarily has its roots in the brainstem and hypothalamus in intense arousal dependent on the midbrain reticular formation (Terpou et al., 2019) and the pontine locus coeruleus (Liddell et al., 2005) and in basic affects such as fear and rage and grief (Panksepp & Biven, 2011) and shame (Corrigan & Elkin-Cleary, 2018).

## Case Study

### Ethical Considerations

The patient has given written informed consent to publish these case details. Permission to report patient data was obtained verbally by the participant, who was fully informed about the purposes of this case report. The participant was informed of how their data would be used and stored, using a paper copy of a written participant information sheet which was provided one week before verbal consent was collected.

### Presenting Problem, Symptom Onset and Previous Treatments

Quentin (a pseudonym) has been in treatment for ten years, having initially presented with avoidance of social interaction and a worsening of anxiety and somatization symptoms. He received a diagnosis of anxiety disorder with panic attacks. The onset of symptoms could be traced back to an exam failure, after which he dropped out of school in a state of growing anxiety. Somatization and derealization symptoms reached their peak, and the first panic attack occurred two years before the presentation.

After the first six months of psychotherapy, based on SCID-II and other clinical features, the diagnosis of avoidant personality disorder in comorbidity with a complex posttraumatic stress disorder (cPTSD) was made. Four years after the first evaluation, the main diagnosis changed because the patient met the criteria for DSM-IV depersonalization disorder (American Psychiatric Association, 1994). Over ten years of psychotherapy treatment, he took antidepressant, antipsychotic and benzodiazepine medication during three different periods without any long-term benefits, in line with what has been documented in the literature (Sierra, 2008; Simeon & Abugel, 2006). Depersonalization symptoms appeared the first time when he was 16–17 years old, in line with the majority of cases that describe the onset of the condition between the ages of 15 and 19 (Baker et al., 2003; Simeon et al., 2003). We report brief descriptions of symptoms made by the patient during some sessions.

*Patient: After hearing my words, the reaction was…you know how when one looks at himself in the mirror and says "but am I that one?!" and the same is when I'm hearing myself talking out loud "am I the one speaking?" it is always the same story that I do not recognize my voice, as if I were a duck—this is my distorted perception… I mumble the words and then my mind says "how does he understand you?" the interlocutor…this is a strong thing… and then I feel disgusted by myself…you hear yourself and say "what I'm doing, what am I saying?" I do not realize […] when you hear it—it is as if you are entering something else, the reality…it seems that this has not happened to me, something that does not belong to me […] too many annoying things… you understand nothing…you know, however, I'm annoying, I annoy myself, of course.*

*Patient: I know I am listening, but it is as if…you are right, now I am starting to think of those tests (referring to depersonalization questions) and the intensity of the sensation is that…*

*Therapist: What?*

*Patient: I feel out of my body… I was looking at myself from the outside. I was looking at the mirror and could not see myself.*

*Therapist: Is it possible that the sensation changes, and therefore, depending on the moment in which you answer the questions, is the score different?*

*Patient: I am sorry to see myself like this… right now all the sensations are coming to me […] I can eat but it is as if the surrounding is not there. It is as if I were in a double place […] I am… it is as if… (accompanying the report with a gesture of the hands to offer me his head) it is as if I took my head and gave it to you.*

*Therapist: Do you feel your head detached?*

*Patient: It is like I do not have it… I cannot visualize anything. It is as if there is a body without a head. Certainly, you think of madness like this (the patient continues to touch his forehead and tries to focus on objects).*

In line with Teicher and Samson (2013), two kinds of emotional maltreatment seem to be connected to pathological mechanisms within the patient's family: verbal abuse and manipulation. Specifically, to serve the emotional needs of his parents, Quentin was placed during childhood in a situation intended to elicit shame and guilt. One marker connected to parent verbal aggression is introjected thinking that continuously criticizes his actions.

Quentin's father is verbally abusing him. He tends to scold and yell at him, demeaning, ridiculing and continuously criticizing his behaviour. His mother makes him feel guilty for his unwell state and for the problems he creates within the family unit. Below are a few examples of our reporting on the patient's fear of failing in life and that his parents might become aware of his symptoms.

*Patient: "After the exam failure I felt as if the word was collapsing upon me…it was like the awareness of the future for me…emptiness […] now I have no more opportunity to do anything, what will I do?—for me everything ended there…what future will I have?—You do not want to work, you do not want to study, how do you finish it? If you do not lean on your family, you end badly or you end up in some institution!" (he is critic of himself in the way he says it).*

Somatization symptoms reach their peak, and the first panic attack occurs in the summer two years before the first visit. A couple of hours after dinner, he lies down on the bed, and after watching a movie, he begins to feel a strong stomachache.

*Patient: "I could not sleep, I was going crazy, I was moving, and I could not stay in bed".*

The main symptoms are stomach pain, sweat, rapid heartbeat and vomiting. The patient gets up and starts walking around the house without being able to stop. A few hours later, he was hospitalized.

The patient tries in every way to hide his illness. Once his parents realized what happened, his anxiety levels increased.

*Patient: "While they were taking me to the hospital, I was lying in the car, and I could no longer move my arms and legs… I did not feel them…while it happened to me, I said to myself—'I'm dying… I'm dying'—but it was impossible that I was dying because I was ironic saying—"damn I'm missing the football match" […] it is as if at that moment you do not feel your body, as if something goes out… but this is impossible because you are super on as if you no longer feel your body… this is… it is as if the body does not belong to you here!! You do not control the body anymore… I do not know how to describe it… by not controlling your body you become a being… a jester (…) it is as if you say—“hand, take that”—and instead he does not take it and therefore you feel…you say—"what is happening to me, something is not working anymore"—and then you worry even more and it takes your whole body. I could not move anymore!".*

Over the years, the patient has been treated with cognitive therapy for social phobia (Clark & Wells, 1995), interpersonal metacognitive treatment for personality disorders (Dimaggio et al., 2015) and phase-oriented intervention using EMDR for complex PTSD (Gonzales & Mosquera, 2012). Despite the improvement in the levels of metacognitive functioning, levels of physiological activation remained high, with difficulty regulating the body state. Constant symptoms of depersonalization and social withdrawal grew in the period after these symptoms.

Talking therapies were not effective in decreasing depersonalization symptoms and improving the patient's self-regulation over a long period. Indeed, trauma impacts the brainstem-level somatic sensory processing mechanisms, and neocortically targeted therapeutic approaches alone cannot reach the lowest brain levels when the traumatic sensory information is stuck and cannot be integrated (Kearney & Lanius, 2022). Between the sensorimotor approaches to psychotherapy, DBR has shown promise as a technique in the treatment of PTSD and associated symptomatology (Kearney et al., 2023), and it was integrated within the phase-oriented intervention to work on the brainstem mechanisms at the basis of depersonalization.

Before starting DBR treatment and at the three time points, Quentin completed a set of measures to assess depersonalization and other comorbidity symptoms. These included the following:

- Cambridge Depersonalization Scale (CDS) (Sierra & Berrios, 2000). The CDS is a 29-item self-report questionnaire designed to measure the frequency and duration of depersonalization symptoms during a period of 6 months. Each CDS item is rated on a two-point Likert scale that measures the frequency and duration of the experience. The sum of frequency and duration defined an index of item intensity (range between 0–290). The Italian version (CDS-IV) (Migliorini et al., 2012) showed high internal consistency and reliability.
- Dissociative Experiences Scale II (DES-II). The DES-II is a 28-item self-report measure with good validity and reliability (Bernstein & Putnam, 1986; Carlson et al., 1993) used in over one hundred published studies (Van Ijzendoorn & Schuengel, 1996). Items are rated on a scale from 0 to 100% of the time. Mean scores below 30 are indicative of low levels of dissociation, while scores above 30 represent high levels of dissociation. For the Italian version of the DES, Schimmenti et al. (2010) found good internal consistency, good test–retest reliability, and good convergent validity in a clinical and nonclinical mixed sample of 600 subjects.
- Self-Report Liebowitz Social Anxiety Scale (SR-LSAS). The SR-LSAS is a 24-item rating scale for the assessment of social anxiety (Liebowitz, 1987). The Italian version of the SR-LSAS showed good criterion and construct validity in patient and nonpatient populations (Baroni et al., 2019).
- World Health Organization Quality of life Abbreviated-version (WHOQOL-BREF). The WHOQOL-BREF defines a QoL profile including four domain scores: physical (7 items), psychological (6 items), social (3 items), and environmental (8 items). The Italian version showed good internal consistency ranging from 0.65 to 0.80. Test–retest reliability values were also good, ranging from 0.76 to 0.93 (De Girolamo et al., 2000).

Quentin completed the CDS and the DES-II, and initial scores on these indicated the presence of significant depersonalization symptoms. He scored 52.8 on the Dissociative Experience Scale during the pre-DBR therapy. The patient showed higher scores not only on items related to depersonalization-derealization but also on other items that constitute the DES absorption subscale (DES-A) (Carlson et al., 1991). Chronic depersonalization, as in the case of Quentin, may be associated with other clinical manifestations of dissociation, such as the tendency to absorption. However, other features of pathological dissociation were not so strong as to displace the primary diagnosis.

The Liebowitz Social Phobia Scale and World Health Organization Quality of Life (short version) were used to measure the behavioural and social problems connected to depersonalization symptoms. Table 1 summarizes all the measures at the four assessment points.

**Table 1:** Quentin's pretherapy and six-, twelve-, and eighteen-month therapy scores

Measure	Pre-DBR therapy score	6 months DBR therapy score	12 months DBR therapy score	18 months DBR therapy score
Dissociative Experience Scale (DES-II)	52.8	21.4	18.2	12.8
Cambridge Depersonalization Scale (CDS)	198	66	46	50
Liebowitz Social Anxiety Scale (LSAS) Fear score	64	45	46	37
Liebowitz Social Anxiety Scale (LSAS) Avoidance score	55	45	46	37
World Health Organization Quality of life—BREF (physical health domain score)	6.2	10.8	10.8	14.2
World Health Organization Quality of life—BREF (psychological health domain score)	7.3	9.3	10.6	11.3
World Health Organization Quality of life—BREF (social relationships domain score)	5.3	6.6	9.3	9.3

### Intervention

Therapy was conducted using the DBR protocol **(**Corrigan & Christie-Sands, 2020) and delivered in a private outpatient service specialized in the treatment of dissociative disorders. Therapy consisted of weekly individual sessions. It was not always possible to work with DBR because of the patient's internal conflict. In that case, the main goal was to focus on interpersonal mechanisms to strengthen the therapeutic alliance to use DBR (Liotti, 2017).

Quentin had already worked on grounding and other techniques in previous years, so these techniques were used as usual within the sessions. Since the second year of therapy, his primary goals for treatment were to reduce his depersonalization symptoms and social phobia symptoms. Even if he had lost hope, he showed his desire to improve his quality of life.

Within the 18-month intervention, he attended 43 DBR sessions with three-time points after 6, 12 and 18 months. The six steps are described in Table 2.

**Table 2:** Standard DBR treatment protocol phases—The O-T-(Shock)-A sequence (Corrigan & Christie-Sands, 2020)

**Step 1. Identifying the Activating Stimulus.** The patient identifies a source of distress to work on. This may be a recent event – for example, as a way into recurring relational difficulties – or something from the past If possible, the core of that distressing experience is identified. For example, a critical look, angry words, humiliating dismissal, a sense of loss or abandonment, a threatening expression or action, the moment that disaster struck – whatever has most actively captured the attention and comes readily to mind
**Step 2. Grounding in the “Where Self”—and the release of tension from the head and neck area.** The patient is asked to let go of their attention on that distressing experience and, instead, to focus, in a number of specific ways, on where the body is situated in this moment Then, “To obtain as much information as possible from the head and neck area, let go of any tension – as much as you can – from the head, neck and shoulders. Let as much ease as is possible come into those muscles.”
**Step 3**. **Orienting to the Activating Stimulus:** “Now notice what happens in those muscles of the forehead, around the eyes, or in the back of the neck when you think of… “ (the selected cause of distress). The therapist delivers as brief and focused a stimulus as possible for this. For example, “when you think of that angry person’s face”; “when you think of that traumatic episode”
**Step 4. Identifying the Orienting Tension**: The essence of DBR is the Orienting Tension so it is necessary to take time to identify this in the muscles of the forehead, around the eyes, or in the back of the neck. If there is nothing from those areas but an immediate move into the affect the client is asked to backtrack to whatever tension came into the body, however fleetingly, before the affect took over awareness. “It is not easy but it would be helpful if you could pick up any tension that passed through the forehead, around the eyes, or the back of the neck before the fear came in....” When the patient has identified the orienting tension there is a need for patience, allowing the processing to move forwards in ultraslow motion. “Stay with that tension in the forehead/around the eyes/at the base of the skull and take your time – no rush to see what comes in next.” There may be a preaffective *shock* evident in a high energy impact on the body, e.g. with bracing of the shoulders or a shiver, shudder, jolt, hollowing, or pressure behind the eyes. If shock is identified there will be a need for therapist and client to sit with this, giving it space and time to clear, before moving onto the affect
**Step 5. Affect**: The sequence can develop slowly to allow the affect – fear, rage, grief, shame – or emotional pain – to come in. When there is a very clear O-T-(Shock)-A sequence processing can flow in its own way. Often memories linked by the sequence common to them will come in. DBR ensures that there is an anchor in the orienting tension that will prevent overwhelm and dissociation If the affect feels stuck or unbearable, release breathing – long, slow outbreaths – can assist in regulating the distress
**Step 6. New Perspective.** When the processing has completed – as evidenced, for example, by the clearing of body distress – the patient can be asked for a new perspective. “Is there any change in how you see yourself as a result of the work you have just done?” This can be associated with any change in body position, especially of the head, and the patient can be encouraged to stay with that after the session to allow the changes to become more established and beneficial in the long-term

In DBR treatment of shock, the aim is to pick up the body’s reaction before there is conscious awareness of emotions or thoughts. An activating stimulus related to the trauma history, for example, a memory of being subjected to verbal abuse, would be used as the starting point. Before this is presented there is preparation through grounding in the “Where Self”, hypothesized to be the egocentric centre of awareness of where the body is in relation to all that is around it, and release of tension in the muscles of the head and neck. The activating stimulus is then presented in a way which will elicit the orienting tension in the muscles of the back of the neck, the forehead, or around the eyes. The orienting tension then functions not only as an anchor to prevent overwhelm but also opens the relevant information file. When there is shock there is usually a bracing of the shoulder muscles and fleeting sensations through the body. The aim in DBR is to slow down this sequence so that the shock can process before the affects come in (see Corrigan & Christie-Sands, 2020).

The clinical use of DBR is more fully described in Kearney et al. (2023) which provides a preliminary analysis of a randomized controlled trial and shows promising effect sizes. The key skill for the processing of shock is the ability to slow down the process to the degree that the orienting tension is separated out from the shock which follows within a fraction of a second. The activating stimulus, for example, a present-day experience of verbal abuse or a memory of an episode of being verbally abused, elicits the orienting tension in the forehead, the muscles around the eyes, or the back of the neck. The therapist is then directive in slowing down the subsequent monitoring of the body’s activation to identify the fleeting shock response. As well as bracing of the shoulders and backward rotation of the head (often so slight as to be almost imperceptible) there may be a fleeting energy, shiver, jolt, hollowing, emptying, or pressure behind the eyes. When these are identified, the patient is encouraged to stay with those shock sensations to give them time and space to dissipate. The therapist’s presence with the patient during identified moments of shock may facilitate the clearing of the shock. Thereafter, the basic affects of fear, rage, grief, and shame emerge, perhaps with episodic memories, and these can be expressed and acknowledged as they process spontaneously.

### Results

#### Measure

**Fig. 1:**
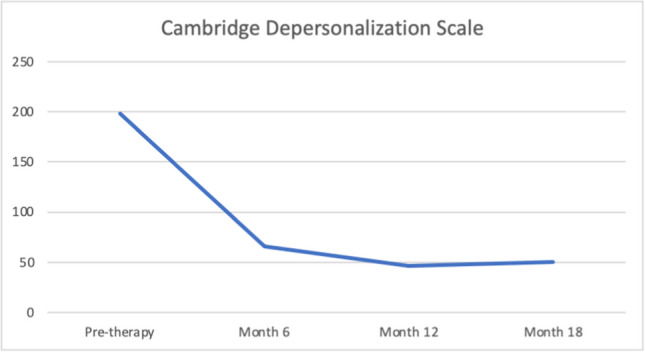
Quentin's pre, six, twelve, and eighteen CDS scores

**Fig. 2:**
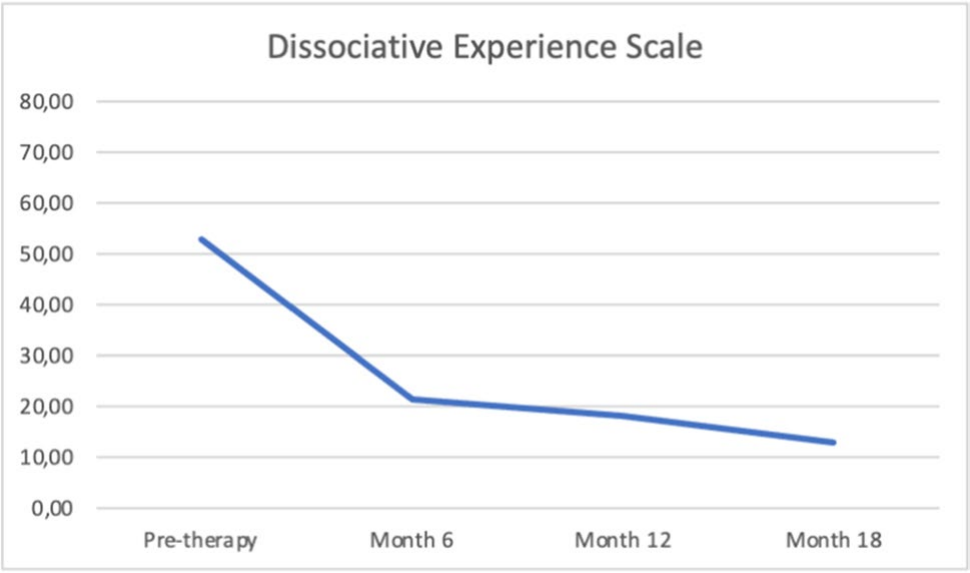
Quentin's pre, six, twelve, and eighteen DES scores

Quentin's scores on the measure of dissociative symptoms decreased from baseline to eighteen months after the start of DBR. Specifically, CDS's depersonalization score decreased sharply after six months of therapy as well as a general reduction in dissociative symptomatology measured by the DES (see Figs. 1, 2). The DES has a cut-off score 20 that is used to identify pathological dissociation. Scores above 20 are generally associated with a diagnosis of dissociative disorder according to DSM-5; lower scores are frequently found in both healthy subjects and psychiatric patients in general (Bernstein & Putnam, 1986). In the CDS a cut-off of 70 has been used to distinguish depersonalization disorder from panic disorder, generalized anxiety disorder and temporal lobe epilepsy (Sierra & Berrios, 2000).

**Fig. 3:**
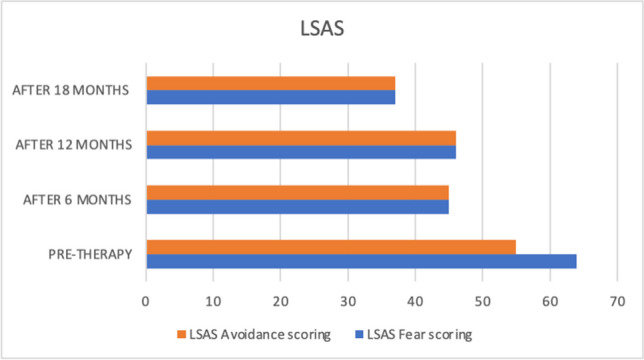
Quentin's pre, six, twelve, and eighteen LSAS scores

**Fig. 4:**
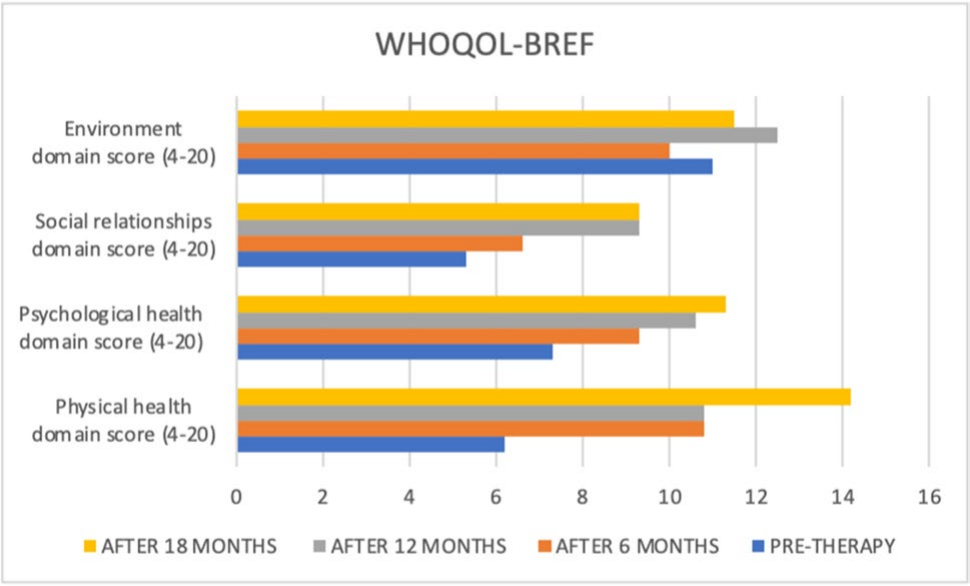
Quentin's pre, six, twelve, and eighteen WHOQOL-BREF scores

Additionally, improvements were found on the LSAS and (WHOQOL-Bref). The social anxiety symptoms decreased after six months, and the score was stable after one year of DBR therapy and decreased again after eighteen months (see Fig. 3). The psychological health and social relationships domain scores of the WHOQOL showed improvements after six, twelve and eighteen months (see Fig. 4).

#### Overall Outcomes

In the year after the therapy described Quentin reduced the dysfunctional interactions with his parents and had no major crises. He reported being more able to cope with physiological symptoms, to self-regulate emotional distress and there was a significant improvement in his quality of life. During that year, Quentin went out alone and sometimes met another person with whom he could talk without feeling depersonalized. He was less phobic of his inner state and was able to continue the work on the core mechanism at the basis of DP disorder.

### Discussion

In 2013, Somer and colleagues systematically reviewed evidence-based treatment for depersonalization-derealization disorder. They discussed how there was no evidence to support the efficacy of psychopharmacology as well as other psychological interventions for treating this psychopathology. They also claimed that further research on the pathogenesis and treatment of depersonalization-derealization disorder was needed (Somer et al., 2013).

The aim of this case was to investigate how feasible, acceptable, and useful DBR is for young people with a diagnosis of depersonalization-derealization disorder. Positive outcomes were achieved after 18 months of DBR therapy. The outcome measures demonstrated clinically significant reductions in the measure of depersonalization symptoms: the use of the LSAS indicated a reduction in social anxiety, while the WHOQOL-Bref indicated improvements in physical health, psychological health, and social relationships. The findings of this case report indicated that DBR therapy may be useful for reducing depersonalization symptoms in a chronic DDD and improving patient quality of life after 43 DBR sessions.

### Limitation

There are some limitations to this single case study. First, the therapist administered all the outcome measures. Accordingly, the scores are more at risk of bias because of the therapeutic relationship and the need by the patient to please the therapist. Likewise, the patient might wish not to betray expectations about the therapy results.

Moreover, there is not a control group to support the conclusion that the changes seen were down to the therapy alone. A further issue relates to the work already done in previous years to create a therapeutic alliance with a patient who tried to avoid any emotional work. This raises questions about what minimal level of body regulation and awareness is necessary to reach before starting with the DBR protocol in patients with depersonalization disorders who can be phobic of their inner states.

This case study does not include any neurobiological measurement to substantiate the assertions of the underlying neurobiological mechanisms of depersonalization. However, this is not the focus of the present paper. Using a single case design (Lobo et al., 2017), this paper aims to illustrate a putative mechanism underpinning the DDD and provide preliminary evidence about the feasibility and effectiveness of reducing depersonalization symptomatology in this complex disorder. The reduction in symptoms could be traced back to other cognitive interventions or the common factors of psychotherapy (Cuijpers et al., 2019). However, this does not appear to be the case, as after more than ten years of treatment, depersonalisation and dissociative symptoms began to decrease with the use of DBR over 18 months.

Future research could involve a larger feasibility study on the use of the DBR for people with DDD within the phased-oriented treatment for dissociative disorders (International Society for the Study of Trauma & Dissociation, 2011). A more extensive trial is required to compare outcomes with those of patients receiving usual treatment for this disorder.

## Conclusion

In this paper, we hypothesized a specific pathogenetic mechanism related to depersonalization, and we proposed a technique to treat this complex symptomatology. A single case report cannot prove that Deep Brain Reorienting intervention was responsible for the changes in depersonalization symptoms, but this did seem to be the case clinically.

To demonstrate conclusively that the improvement is due to deep brain reorienting therapy, randomized, controlled prospective studies will have to be conducted in the future and, ideally, linked with neuroimaging assessment of the putative mechanisms of change.

## Data Availability

The dataset generated during the current study is available from the corresponding author, on reasonable request.
